# Testing the efficacy of an HIV stigma reduction intervention with medical students in Puerto Rico: the SPACES project

**DOI:** 10.7448/IAS.16.3.18670

**Published:** 2013-11-13

**Authors:** Nelson Varas-Díaz, Torsten B Neilands, Francheska Cintrón-Bou, Melissa Marzán-Rodríguez, Axel Santos-Figueroa, Salvador Santiago-Negrón, Domingo Marques, Sheilla Rodríguez-Madera

**Affiliations:** 1 Center for Social Research, University of Puerto Rico, Río Piedras Campus, San Juan, Puerto Rico; 2 Center for AIDS Prevention Studies (CAPS), University of California, San Francisco, CA; 3 Clinical Psychology Program, Ponce School of Medicine and Health Sciences, Ponce, Puerto Rico; 4 Social Sciences Department, Graduate School of Public Health, University of Puerto Rico, Medical Sciences Campus, San Juan, Puerto Rico

**Keywords:** HIV, stigma, intervention, reduction, Puerto Rico

## Abstract

**Introduction:**

Stigma associated with HIV has been documented as a barrier for accessing quality health-related services. When the stigma manifests in the healthcare setting, people living with HIV receive substandard services or even be denied care altogether. Although the consequences of HIV stigma have been documented extensively, efforts to reduce these negative attitudes have been scarce. Interventions to reduce HIV stigma should be implemented as part of the formal training of future healthcare professionals. The interventions that have been tested with healthcare professionals and published have several limitations that must be surpassed (i.e., lack of comparison groups in research designs and longitudinal follow-up data). Furthermore, Latino healthcare professionals have been absent from these intervention efforts even though the epidemic has affected this population disproportionately.

**Methods:**

In this article, we describe an intervention developed to reduce HIV stigma among medical students in Puerto Rico. A total of 507 medical students were randomly introduced into our intervention and control conditions.

**Results:**

The results show statistically significant differences between the intervention and control groups; intervention group participants had lower HIV stigma levels than control participants after the intervention. In addition, differences in HIV stigma levels between the groups were sustained for a 12-month period.

**Conclusions:**

The results of our study demonstrate the efficacy of the modes of intervention developed by us and serve as a new training tool for future healthcare professionals with regard to stigma reduction.

## Introduction

### The burden of HIV in the Caribbean and Puerto Rico

The Caribbean is the second most HIV-affected region in the world with an estimated prevalence of 1% [1]. The island of Puerto Rico, a non-incorporated territory of the United States with a population of 3.7 million, has been heavily affected by the epidemic with more than 40,000 reported infections [2, 3]. Puerto Rico holds the fifth position in AIDS diagnosis rates (26.4/100,000) in the United States and the fourth position in its prevalence among those older than 13 years (335.1/100,000) [4]. A mainly male-driven epidemic (74%), the most common modes of transmission of HIV are needle sharing for illegal drug use (45%), unprotected heterosexual contact (27%), and unprotected relations between men who have sex with men (17%). Recent research has documented that an estimated 1% of the Puerto Rican population is living with HIV [5].

### HIV stigma and its consequences

HIV stigma remains one of the most challenging barriers to maintaining the overall health of people living with HIV (PLHIV). The stigma affects mental health by fostering depression, low self-esteem and anxiety [6, 7]. It also influences physical health by hindering adherence to antiretroviral treatment, accelerating disease progression [8, 9]. Finally, HIV stigma has been shown to hinder social interaction because PLHIV can feel ostracized, which leads to significant reduction in or complete elimination of their social networks [10].

The consequences of HIV stigma worsen when stigmatizing behaviour originates from people who are important in the lives of PLHIV, such as healthcare professionals [7]. This is particularly true for physicians, who play such a pivotal role in treatment. These professionals represent the first line of contact for treatment and the basis of knowledge of effective strategies to restrain disease progression. They can also be an important source of support for PLHIV [11]. When physicians stigmatize PLHIV, access to effective treatment can be limited. Previous studies carried out in Puerto Rico have documented how healthcare professionals, particularly physicians, manifest HIV stigma [12]. Because physicians play an essential role in the lives of PLHIV in Puerto Rico, scientifically tested strategies to reduce stigma among them are urgently needed.

A review of the published literature on HIV stigma reduction efforts reveals important gaps that must be addressed, including lack of efficacious interventions to reduce stigma among healthcare professionals from Latino backgrounds [13, 14].

### HIV stigma reduction interventions with health professionals

Reducing HIV stigma in healthcare scenarios has been a public health concern in multiple countries [10, 11, 15–20]. These efforts have pointed towards the need for developing interventions that place emphasis on the individuals, facility environments and policies [16, 21].

A review of published scientific studies on HIV demonstrates that existing interventions to reduce HIV stigma are scarce, and those that focus specifically on physicians are few [22]. This gap is worsened by the unsystematic use of measures to evaluate HIV stigma reduction and problems with internal validity of research designs. These challenges were initially posed in a meta-analysis published by Brown and colleagues [13], which yielded only 22 published articles documenting scientifically tested interventions. Only 5 of the 22 interventions were developed specifically for healthcare professionals. Some of the limitations identified are: (1) using small samples, (2) conducting interventions that did not reduce fear of PLHIV, (3) not measuring HIV stigma with reliable and valid scales, and (4) seeing little evidence of sustained intervention impact beyond three months.

A recent intervention carried out in China has addressed structural changes by making universal precaution supplies accessible in hospitals and documented significant differences in attitudinal and behavioural changes of its participants [23–25]. Although such efforts are encouraging and scientifically sound, similar strategies have not been implemented with Latino health professionals. Considering that HIV has impacted the Latino community disproportionately, including Puerto Ricans, the need to develop interventions to reduce HIV stigma among physicians caring for Latinos is urgent [12].

### The SPACES intervention

The developed intervention is based on extensive qualitative work with healthcare professionals and medical students in Puerto Rico [12, 26–28]. We named our intervention SPACES in line with our promotional tagline of fostering “stigma-free spaces in medical scenarios.” The intervention is a nine-hour workshop divided into three sessions (three hours each). SPACES addresses the sources and functions of HIV stigma based on Goffman's theoretical contributions [29], issues that can worsen its consequences based on Jones’ stigma dimensions [30] and focuses on both instrumental and symbolic stigmas manifested for HIV [31, 32]. A description of the content for each session can be found in Table 1.

**Table 1:** Overview of the SPACES intervention

Session	Content and educational technique
1	Content: Information on HIV stigma and its consequences on service delivery. Educational technique: In this session, we addressed participants’ knowledge of HIV epidemiological data in Puerto Rico. Small groups were asked to outline the social groups that were most impacted by the epidemic. We later contrasted their perceptions of impacted groups with actual epidemiological data. This process allowed us to discuss how HIV stigma can influence medical students’ perceptions of the epidemic and the role stigma plays in this process. Furthermore, we discuss how social stigma related to HIV is also intertwined with other pre-existing stigmas related to illegal drug use, homosexuality and gender roles.
2	Content: The role of negative emotions in HIV stigma. Educational technique: In this second session, we addressed the role of negative emotions in fostering HIV stigma attitudes and behaviours when interacting with PLHIV. Participants were exposed to clinical vignettes of HIV infection cases and asked to complete charts detailing the types of emotions they experienced when discussing the cases (e.g., fear, shame, disgust, admiration). Small groups discussions on these cases were complemented with whole group sessions in which the role of emotions in stigma was explained. Culturally accepted emotions such as “pity” were discussed as potential sources of stigma.
3	Content: Skills for stigma-free interaction with PLHIV. Educational technique: In this last session, we discussed specific behavioural skills for interacting with PLHIV in clinical scenarios in a non-stigmatizing manner. Furthermore, we discussed how HIV stigma that is manifested in society could also be manifested in clinical encounters by both the physician (i.e., denying services, providing sub-standard services) and PLHIV (i.e., low self-esteem, self-stigmatizing attitudes). Examples of stigma manifestations through media outlets and policies were discussed in order to provide an overview of a social scenario in which clients might feel stigmatized. We stressed the importance of providing stigma-free spaces and interactions in medical settings.

The SPACES workshops were offered to students as extracurricular activities to ensure that their ongoing classwork and clinical practice were not affected. To facilitate attendance and participation, the workshops were provided within the students’ medical schools. Participants were provided a stipend each time they completed a questionnaire for a total sum amount of $125 ($25 at T1–T3 and $50 at T4). The workshops were facilitated by six health professionals with advanced degrees (MA and PhDs) and previous experience with HIV-related patients. As part of our process evaluation, participants mentioned that the intervention allowed them to correct information about HIV. One participant mentioned: “It was very important because some of the information I had was totally wrong.” They also described the intervention as a positive experience, and another participant reported the following: “I feel more open minded to working with patients.” Our study aimed at assessing the efficacy of the SPACES intervention in reducing HIV stigma attitudes among medical students in Puerto Rico.

## Methods

To achieve the aim of our study, we implemented a randomized controlled trial with group randomization to the SPACES intervention and a non-stigma control group. Details are presented below.

### Participants

Our sample consisted of 507 second year medical students. The sample characteristics are described below in the results section.

### Procedure

Participants were recruited from the four largest medical schools in Puerto Rico. Our team visited medical schools throughout the Island to meet with students and invite them to participate. Their participation was voluntary and we ensured them that it would not influence their evaluation by other professors in their courses. Groups of 20 were randomized into our intervention and control conditions. We implemented a basic HIV epidemiology workshop as a time- and attention-matched control group experience. Participants completed our baseline measure (T1) before engaging in the workshops and immediately after (T2) completing the third and last sessions. They were contacted by phone and via email in order to complete the 6- (T3) and 12-month follow-up (T4) over the web. Attendance to our intervention workshops was high with 86% of participants completing all three sessions, 10% completing two sessions and 4% completing one session. Drop-out rates at T2 and T3 measurements were low, with 92% of the participants completing T2 measures and 85% completing T3. A total of 385 out of the 507 participants completed the T4 measure with a 24% attrition rate. We implemented our intervention from January, 2008, through April, 2011.

### Measures

Participants completed a self-administered questionnaire containing several scales, including HIV knowledge, perceptions of self-efficacy for providing services, social desirability and HIV stigma, the primary outcome variable. The Spanish HIV Stigma Scale (SHASS) is a reliable and culturally appropriate scale previously developed in Puerto Rico, which measures 11 dimensions of HIV stigma: 1) restriction of PLHIV's rights, 2) PLHIV obliged to reveal HIV status, 3) responsibility of PLHIV for their HIV infection, 4) lack of productivity of PLHIV, 5) personal characteristics of PLHIV, 6) fear of infection, 7) emotions associated with HIV, 8) closeness to death, 9) need to control PLHIV, 10) PLHIV as vectors of infection and 11) body signs of HIV. All items are measured by a 5-point Likert-type scale ranging from strongly agree (5) to strongly disagree (1) [33].

### Data analysis

*Descriptive Analyses –* Initial descriptive analyses characterized the sample using one-way and cross-tabular frequency tables with counts and percentages displayed by control versus intervention group. Likelihood ratio chi-square tests were used to compare percentages across groups on unordered categorical variables. Mantel-Haenszel chi-square tests were used to test for control versus intervention group differences on the following ordinal variables: importance of religion, income, risk for HIV infection and perceptions of medical students’ attitudes towards PLHIV. Exact chi-square test statistics were substituted for the default asymptotic chi-square tests, if the expected cell counts were less than five. Means and standard deviations were generated for the HIV-stigma measure for each group at each measurement wave.

*Comparisons of Means –* The primary analysis was a comparison of means performed using a 2 (Group: Intervention vs. Control) by 4 (Wave: 1, 2, 3 or 4) repeated measures analysis. Group and Wave were treated as fixed effects. The covariance structure among the repeated measurements was set to unstructured as recommended by Diggle et al. (2002) [34] for designs with few fixed measurement points. Model-based means were estimated and compared using restricted maximum likelihood (REML) in SAS PROC MIXED with the Kenward-Roger method used to compute the denominator degrees of freedom [35]. The Group-by-Wave interaction omnibus test and its constituent components were used to determine whether the differences between the intervention and control groups differed over time. These tests were followed up with paired comparisons of the intervention and control group means within each of the four waves of measurement; these paired comparisons’ *p*-values were adjusted using Sidak's method to control the Type 1 error rate [36]. Cohen's standardized effect size *d* was computed for the post-intervention mean differences as an index of the magnitude of the intervention effect.

## Results

*Sample Characteristics –* The sample was approximately gender balanced, heterosexual and Puerto Rican (Table 2). Nearly half the sample (46%) had tested for HIV and none reported being HIV-positive. However, more than one-fourth of the sample knew someone with HIV and the vast majority reported that HIV was discussed in their medical school coursework, yet more than 90% of the participants believed other medical students discriminated against PLHIV. Approximately two-thirds indicated religion as being important or very important, and 80% either disagreed or felt unsure that they were prepared to provide services to PLHIV (Table 2).

**Table 2:** Sample characteristics

	Intervention		Control			
				
Variable	*N*	%	*N*	%	χ^2^ (DF)	*p*
Male gender	123	45.4	109	46.2	0.03 (1)	0.86
Heterosexual orientation	264	97.8	233	98.7	0.67 (1)	0.51
National origin					7.69 (3)	0.07
Puerto Rican	213	78.9	204	86.4		
Dominican	1	0.4	3	1.3		
Cuban	10	3.7	5	2.1		
Other	46	17.0	24	10.2		
Ever tested for HIV	125	46.3	108	46.4	0.0002 (1)	0.99
HIV negative test result (among those who tested)	123	96.1	99	94.3	0.42 (1)	0.55
Knew someone with HIV	79	29.7	61	26.9	0.48 (1)	0.49
Taken a class where HIV was discussed	239	88.2	196	83.4	2.39 (1)	0.12
Believe other medical students discriminate	245	90.7	216	92.3	0.40 (1)	0.53
Religion importance						
Not important	29	10.7	18	7.6	0.67 (1)	0.41
Somewhat important	69	25.6	57	24.2		
Important	89	33.0	90	38.1		
Very important	83	30.7	71	30.1		
Annual income						
<$10,000	81	31.6	89	39.6	0.08 (1)	0.77
$10,001–$20,000	30	11.7	17	7.6		
$20,001–$30,000	26	10.2	16	7.1		
$30,001–$40,000	37	14.5	23	10.2		
$40,001–$50,000	30	11.7	15	6.7		
$50,001–$60,000	8	3.1	12	5.3		
>$60,000	44	17.2	53	23.6		
Perception of risk of HIV infection						
Not at all	73	27.2	56	23.9	2.96 (1)	0.09
A little	150	56.0	116	49.6		
A regular amount	31	11.6	52	22.2		
A lot	14	5.2	10	4.3		
Medical students attitudes towards PLHIV						
Totally positive	12	4.4	12	5.1	0.26 (1)	0.61
Partially positive	72	26.6	56	23.8		
Neutral	123	45.4	106	45.1		
Partially negative	64	23.6	61	26.0		
Prepared to provided services to PLHIV						
Totally agree	13	4.8	7	3.0	1.40 (1)	0.24
Partially agree	42	15.5	39	16.5		
Undecided	178	65.7	146	61.9		
Partially disagree	38	14.0	44	18.6		

Notes: Percentages and *N*s will not always sum to 100% due to small amounts of missing data. For sexual orientation, the comparison category is homosexual/lesbian/bisexual. For HIV testing, the comparison group was “Don't know” (no respondents reported an HIV-positive test result).

*Comparisons of Means –* Statistically significant main effects for intervention group (*F*(1, 499)=14.97, *p=*0.0001) and wave of measurement (*F*(3, 439)=22.23, *p*<0.0001) were found. These main effects were qualified by a statistically significant Group-by-Wave interaction (*F*(3, 439)=8.82, *p<*0.0001). The three individual components of the interaction effect were then examined to determine whether the mean differences between the groups at baseline were statistically different from the same group differences at each follow-up point, with the difference between the two differences quantified as *D*. The comparison of the baseline group difference with the immediate follow-up group difference was significant (*D*=0.16, *t*(472.7)=4.91, *p*<0.0001). The comparison of the baseline group difference with the six-month group difference was also significant, though the effect was weaker (*D*=0.08, *t*(460.8)=2.18, *p*=0.03). Finally, the comparison of the baseline group difference with the 12-month group difference was also significant (*D*=0.12, *t*(431.5)=2.90, *p*=0.004). Follow-up paired comparisons of the group's means within each time point revealed no significant difference between the groups at baseline, but the mean levels of HIV stigma were significantly lower in the intervention group at each of the three follow-up measurement waves (see Table 3). The Cohen's *d* value for the comparison of intervention and control group means immediately following the intervention was −0.40. At the six month follow-up, the corresponding *d* value was −0.29, and at the 12 month follow-up, the *d* value was −0.30. Benchmark values for *d* are *d*=0.20 for a small effect and *d*=0.50 for a medium effect. Therefore, the intervention exhibited an effect between small and medium in reducing HIV stigma (see Table 4).

**Table 3:** Means and differences for HIV stigma scores

	Sample means (SD)								
				
	Control			Intervention					
					
Wave	*N*	*M*	*SD*	*N*	*M*	*SD*	*t*	*df*	*p*
1	234	2.88	(0.48)	269	2.79	(0.51)	−1.82	501.5	0.25
2	219	2.83	(0.51)	241	2.61	(0.58)	−4.83	494	<0.0001
3	197	2.80	(0.54)	225	2.64	(0.55)	−3.22	487	0.0055
4	179	2.77	(0.57)	206	2.59	(0.59)	−3.74	473	0.0002

Notes: M=Mean; SD=Standard Deviation. *t*, *df* and *p*-values are estimated from SAS PROC MIXED with the Kenward-Roger degrees-of-freedom method, which features non-integer DF values. *p*-Values for paired comparisons of group means were adjusted for multiple comparisons using Sidak's method.

## Discussion

The results of our study suggest that the SPACES intervention is an efficacious tool for stigma reduction among medical students. Results evidenced significantly lower stigma levels immediately following intervention among persons who completed the intervention, irrespective of starting levels of stigma. Furthermore, significantly lower stigma levels were documented at 6 and 12 months. Therefore, the SPACES intervention is the first stigma reduction effort to be systematically tested in Puerto Rico via a randomized controlled trial with demonstrated stigma reduction.

**Table 4:** Model-implied means and differences from latent growth model analysis

	At mean baseline stigma					
		
Wave	Control	Intervention	*D*	*Z*	*p*	*d*

1	2.83	2.83	0	–	–	0
2	2.80	2.63	−0.17	−6.69	<.001	−0.30
3	2.79	2.68	−0.10	−3.33	<.001	−0.15
4	2.75	2.61	−0.14	−3.30	.001	−0.15
	At 1 SD below baseline stigma					
		
Wave	Control	Intervention	*D*	*Z*	*p*	*d*

1	2.39	2.39	0	–	–	
2	2.36	2.10	−0.26	−6.63	<0.001	−0.30
3	2.34	2.17	−0.17	−3.85	<0.001	−0.17
4	2.31	2.08	−0.23	−4.40	<0.001	−0.20
	At 1 SD above baseline stigma					
		
Wave	Control	Intervention	*D*	*Z*	*p*	*d*

1	3.27	3.27	0	–	–	
2	3.25	3.17	−0.08	−2.00	0.045	−0.09
3	3.22	3.18	−0.04	−1.07	0.28	−0.05
4	3.20	3.15	−0.05	−0.98	0.33	−0.04

Notes: *N*=506. *D*=Difference defined as control group mean minus intervention group mean. *d*=approximate standardized *D* defined as *D*/(SE*sqrt(*N*)), where *SE* is the standard error of the estimate and *N* is the sample size. Model-implied means, differences and associated test statistics were estimated using full-information maximum likelihood (FIML) with residual bootstrap-based standard errors in M*plus* 7.

This initial evaluation of the SPACES intervention provides the medical training community with a promising tool for HIV stigma reduction. We understand that several strengths in the process of development and implementation of the program will allow medical schools to easily uptake the SPACES intervention. For example, the SPACES intervention can be incorporated into existing medical coursework on issues of cultural competence and ethical treatment of patients due to its closely interrelated content. Also, the workshops are similar in length and structure to medical classroom activities and therefore could easily be incorporated into existing classwork. Finally, the intervention can strengthen the portfolios of medical schools on evidence-based training of medical students, which is an important aspect of curriculum accreditation. For these reasons, we believe that medical schools are in an advantageous position to incorporate the SPACES intervention into their academic sequences and institutional training policies.

The SPACES intervention also has the potential to address stigma reduction at institutional and government policy levels. For example, institutional policies within medical schools could focus on the need for stigma reduction as part of medical training and integrate SPACES as a stigma reduction tool. This potential policy level decision within medical training scenarios could help to ensure that participation in stigma reduction efforts is not simply voluntary but an integral aspect of medical training in which all students and faculty members should engage. Furthermore, the SPACES intervention is an important initial step in the development of stigma reduction efforts for already practicing physicians, which could benefit from this effort with minor modifications to our existing intervention. This initial effort has the potential to introduce stigma reduction as a vital subject of medical school training and, in the future, accreditations for practicing physicians.

Although these results are promising, and provide HIV stigma reduction practitioners with a new tool for action, several steps need to be taken in the future in order to continue innovating in the field of stigma reduction. Some examples include 1) exploring by which mechanisms the intervention changes attitudes (i.e., mediators) and for whom the intervention is most efficacious (i.e., moderators), 2) tailoring the SPACES intervention to reduce stigma combinations (i.e., homophobia, stigma towards illegal drug users), 3) documenting the sustained effects of the intervention through longer periods of time (i.e., 24 and 36 months) when medical students are exposed to experiences that can foster negative attitudes towards PLHIV and 4) exploring the consequences of stigma attitude reduction on its behavioural manifestations (e.g., denying care, providing substandard care) when interacting with PLHIV.

We understand that some of these recommended steps will guide the development of future stigma reduction interventions in the coming years due to their importance for HIV prevention and treatment. It is of vital importance to continue generating efforts that produce data that demonstrate stigma reduction via a plurality of mechanisms. For example, although the reduction of stigma attitudes has been utilized as an indicator of interventions’ efficacy, we still know very little about the implications of stigma attitude reduction on health professionals’ specific behaviours in the clinical encounter. The behavioural implication of stigma attitude reduction needs to be better explored though interventions that use both attitudinal and observational measurements to assess their joint impact on stigma reduction. In this same line, future stigma reduction interventions need to report their standardized effect sizes, which are seldom included in scientific papers, in order for the field to develop a base rate to which new interventions can be compared.

The stigmatization of HIV continues to be a problem for PLHIV at a global level. Stigma reduction interventions need to be tested and disseminated in order to have them widely available and potentially impact the lives of PLHIV. This entails adopting a global perspective when scaling up stigma reduction interventions. It is our hope that our work in Puerto Rico will serve as a model for future stigma reduction interventions with medical students and that in unison with other tested efforts, we can collectively impact the training of stigma-free physicians.

## References

[1] UNAIDS (2012). Global fact sheet. http://www.unaids.org/en/media/unaids/contentassets/documents/epidemiology/2012/gr2012/20121120_FactSheet_Global_en.pdf.

[2] U.S. Census Bureau (2010). Características Sociales Seleccionadas en Puerto Rico. http://factfinder2.census.gov/faces/nav/jsf/pages/community_facts.xhtml.

[3] Puerto Rico AIDS Surveillance (2013) (2013). Cumulative HIV/AIDS cases diagnosed as of February.

[4] CDC (2010). Epidemiology of HIV infection. http://www.cdc.gov/hiv/topics/surveillance/resources/slides/general/slides/general.ppt#.

[5] Perez CM, Marrero E, Meléndez M, Adrovet S, Colón H, Ortiz AP (2010). Seroepidemiology of viral hepatitis, HIV and herpes simplex type 2 in the household population aged 21–64 years in Puerto Rico. BMC Infect Dis.

[6] Kuang W, Li J, Ma Y, Liao J (2005). Survey on psychologic status and quality of life for HIV infected people or AIDS patients. J Sichuan University.

[7] Li L, Lin C, Wu Z, Rotheram-Borus MJ, Detels R, Jia M (2007). Stigmatization and shame: consequences of caring for HIV/AIDS patients in China. AIDS Care.

[8] Malavé S, Varas-Díaz N (2006). Regulando la enfermedad a través de la definición y la restricción: Profesionales de la salud hablan sobre el VIH/SIDA. Ciencias de la Conducta.

[9] Sayles JN, Wong MD, Kinsler JJ, Martins D, Cunnigham WE (2009). The association of stigma with self reported access to medical care and antiretroviral therapy adherence in persons living with HIV/AIDS. J Gen Intern Med.

[10] Rahmati-Najarkolaei F, Shamsaddin N, Aminshokravi F, Bazargan M, Ahmadi F, Hadjizadeh E (2010). AIDS society: experience stigma in healthcare settings among adults living with HIV in the Islamic Republic of Iran. J Int AIDS Soc.

[11] Hossain MB, Kippax S (2011). Stigmatized attitudes toward people living with HIV in Bangladesh: health care workers’ perspectives. Asia Pac J Publ Health.

[12] Varas-Díaz N, Neilands TB, Malavé-Rivera S, Betancourt E (2010). Religion and HIV stigma: implications for health professionals in Puerto Rico. Global Publ Health.

[13] Brown L, Mcintyre K, Trujillo L (2003). Interventions to reduce HIV/AIDS stigma: what have we learned?. AIDS Educ Prev.

[14] Ho Y, Li X (2009). HIV/AIDS behavioral interventions in China: a literature review and recommendation for future research. AIDS Behav.

[15] Nyblade L, Stangl A, Weiss E, Ashburn K (2009). Combating HIV stigma in healthcare settings: what works?. J Int AIDS Soc.

[16] Mahendra VS, Gilborn L, George B, Samson L, Mudoi R, Jadav S (2006). Reducing AIDS-related stigma and discrimination in Indian hospitals.

[17] Apinundecha C, Laohasiriwong W, Cameron MP, Lim S (2007). A community participation intervention to reduce HIV/AIDS stigma, Nakhon Ratchasima province, northeast Thailand. AIDS Care.

[18] Uys L, Chirwa M, Kohi T, Greeff M, Naidoo J, Makoae L (2009). Evaluation of a health setting-based stigma intervention in five African countries. AIDS Patient Care STDS.

[19] Rutledge SE, Abell N, Padmore J, McCann TJ (2009). AIDS stigma in health services in the Eastern Caribbean. Sociol Health Illn.

[20] Zhang KL, Detels R, Liao S, Cohen M, Yu DB (2008). China's HIV/AIDS epidemic: continuing challenges. Lancet.

[21] Oanh KTH, Ashburn K, Pulerwitz J, Ogden J, Nyblade L (2008). Improving hospital-based quality of care in Vietnam by reducing HIV-related stigma and discrimination, a Horizons final report.

[22] Sengupta S, Banks B, Jonas D, Miles M, Smith S (2011). HIV interventions to reduce HIV.AIDS stigma: a systematic review. AIDS Behav.

[23] Neema S, Atuyambe LM, Otolok-Tanga E, Twijukye C, Kambugu A, Thayer L (2012). Using a clinic based creativity initiative to reduce HIV related stigma at the Infectious Diseases Institute, Mulago National Referral Hospital, Uganda. Afr Health Sci.

[24] Li L, Zunyou W, Li-Jung L, Chunqing L, Jihui G, Manhong J (2013). Reducing HIV-related stigma in healthcare settings: a randomized controlled trial in China. Am J Publ Health.

[25] Li L, Li-Jung L, Chunqing L, Zunyou W, Rotheram-Borus M (2010). HIV prevention intervention to reduce HIH-related stigma: evidence from China. AIDS.

[26] Varas-Díaz N, Neilands TB, Cintrón-Bou F, Santos-Figueroa A, Marzán-Rodríguez M, Marqués D (2013). Religion and HIV stigma in Puerto Rico: a cultural challenge for training future physicians. J Int Assoc Phys AIDS Care.

[27] Varas-Díaz N, Neilands TB, Cintrón-Bou F, Santos-Figueroa A, Rodríguez-Madera S, Santiago-Negrón S (2012). The role of gender on HIV stigma among medical students in Puerto Rico: implications for training and service delivery. Puerto Rico Health Sci J.

[28] Varas-Díaz N, Santiago-Negrón S, Torsten TB, Cintrón-Bou F, Malavé-Rivera S (2010). Stigmatization of illicit drug use among Puerto Rican health professionals in training. Puerto Rico Health Sci J.

[29] Goffman E (1963). Stigma: notes on the management of spoiled identity.

[30] Jones EE, Farina A, Hastorf A, Markus H, Miller DT, Scott RA (1984). Social stigma: the psychology of marked relationships.

[31] Herek GM, Glunt EK (1988). An epidemic of stigma: public reactions to AIDS. Am Psychol.

[32] Herek GM (1999). AIDS and stigma. Am Behav Sci.

[33] Varas-Díaz N, Torsten TB (2009). Development and validation of a culturally appropriate HIV stigma scale for Puerto Rican health professionals in training. AIDS Care.

[34] Diggle PJ, Heagarty P, Liang K-Y, Zeger SL (2002). Analysis of longitudinal data.

[35] Littell RC, Milliken GA, Stroup WW, Wolfinger RD, Schabenberger O (2006). SAS system for mixed models.

[36] Šidák Z (1967). Rectangular confidence regions for the means of multivariate normal distributions. J Am Stat Assoc.

