# Testing the efficacy of an HIV stigma reduction intervention with medical students in Puerto Rico: the SPACES project

**DOI:** 10.7448/IAS.16.1.18973

**Published:** 2013-12-23

Regarding the paper ‘Testing the efficacy of an HIV stigma reduction intervention with medical students in Puerto Rico: the SPACES project’ by Nelson Varas-Díaz, Torsten B Neilands, Francheska Cintrón-Bou, Melissa Marzán-Rodríguez, Axel Santos-Figueroa, Salvador Santiago-Negrón, Domingo Marques and Sheilla Rodríguez-Madera

Published in *Journal of the International AIDS Society Supplement 2*. Citation: http://www.jiasociety.org/index.php/jias/article/view/18670 | http://dx.doi.org/10.7448/IAS.16.3.18670

Table 4 and the table citation were erroneously included and did not actually contain the data that was referred to in the text.

The corrected paper can be found below:
